# Movement smoothness and plasma proteomics are associated with functional impairment in knee osteoarthritis

**DOI:** 10.1016/j.ocarto.2026.100854

**Published:** 2026-07-30

**Authors:** Karol Gawelowicz, Morten Bilde Simonsen, Margareta Hedström, Eva Broström, Cecilia Aulin, Josefine E. Naili

**Affiliations:** a Department of Women's and Children's Health, Karolinska Institutet, Karolinska Vägen 37 A, QA 02:07, 171 76 Stockholm, Sweden; b Department of Highly Specialised Paediatric Orthopaedics and Medicine, Astrid Lindgren Children's Hospital, Karolinska University Hospital, Stockholm, Sweden; c Department of Materials and Production, Aalborg University, Fibigerstræde 16, 9220 Aalborg Ø, Denmark; d Center for Mathematical Modelling of Knee Osteoarthritis, Aalborg University, Denmark; e Department of Clinical Science, Intervention and Technology, CLINTEC Karolinska Institutet, 171 77 Stockholm, Sweden; f Trauma and Reparative Medicine Theme (TRM), Karolinska University Hospital Stockholm, Sweden; g Motion Analysis Lab, Karolinska University Hospital Stockholm, Sweden; h Department of Medicine Solna, Division of Rheumatology, Centre for Molecular Medicine, Karolinska Institutet, Karolinska University Hospital Stockholm, Sweden

**Keywords:** Inertial measurement units, Spectral arc length (SPARC), Biomarkers, Targeted proteomics, PROMs, Performance-based function

## Abstract

**Objective:**

To evaluate clinical outcomes (performance-based tests and patient-reported outcome measures [PROMs]) and biomechanical measures of movement smoothness, and explore their associations with molecular markers in individuals with knee osteoarthritis (KOA).

**Method:**

Thirty-six individuals with KOA (mean age 62.7 years; 75% female) and 19 controls (mean age 62.3 years; 63% female) performed four functional tests: 10 m Fast-Paced Walk (10MWT), 30 s Sit-to-Stand (30STS), Single Leg Mini Squat (SLMS), and Jump for Distance. Movement smoothness during 30STS and SLMS was quantified using Spectral Arc Length (SPARC) from inertial sensors. Individuals with KOA completed PROMs, and pain was assessed in both groups. Proteomic profiling (Olink) identified differentially expressed proteins (DEPs).

**Results:**

Individuals with KOA showed poorer performance across all functional tests (p < 0.001–0.04) and higher pain (p < 0.001). SPARC metrics indicated reduced movement smoothness, with lower mean SPARC (p < 0.001–0.004) and higher variance (p < 0.001). Nine DEPs distinguished the KOA group: one upregulated (DEFB4A) and eight downregulated (FKBP5, CETN2, TBCB, AXIN1, PMVK, PTPN1, PRTFDC1, ST1A1). All downregulated proteins correlated positively with 30STS performance (|r| = 0.37–0.57), and three negatively with 10MWT (|r| = 0.36–0.40). Higher mean SPARC during 30STS correlated positively with these proteins and partially during SLMS. PROMs showed moderate associations with functional outcomes.

**Conclusion:**

Individuals with KOA exhibited impaired function, reduced movement smoothness, and distinct proteomic profiles. Associations between proteins and movement quality suggest a molecular link to neuromuscular function. Combining proteomics with objective movement analysis and PROMs may improve understanding of KOA heterogeneity and support patient stratification.

## 1 Introduction

Knee osteoarthritis (KOA) is a prevalent joint disorder characterised by pain, reduced range of motion (ROM), and muscle weakness [1]. These symptoms often result in functional limitations, restricted ability to perform activities of daily living [2], and reduced physical activity [3]. KOA complexity and heterogeneity have prompted initiatives to better characterise the disease using biological, structural, and functional variables [[4], [5], [6]]. Beyond these domains, biomechanical factors represent another important dimension, as altered joint loading and movement patterns contribute to both structural damage and symptoms [7]. Therefore, integrating biomechanical measures with other variables may provide a deeper understanding of the disease [7,8].

Technological advances facilitate detailed biomechanical data collection using inertial measurement units (IMUs). Kinematic evaluations using few sensors offer a feasible way to enhance understanding of the physical manifestations of KOA. A recent white paper emphasised that kinematic measures provide more subtle and reliable insights than standard parameters (e.g., time, distance, repetitions), offering a stronger basis for evaluating disease progression and therapeutic efficacy [9]. Among the metrics derived from these assessments, movement smoothness, defined as movement continuity or non-intermittency, is considered a hallmark of healthy and well-trained motor behaviour [10]. It is thought to reflect effort minimisation and learned coordinative processes in sensorimotor control [10]. Although not a direct measure of neuromuscular function, reduced movement smoothness, indicating greater intermittency, may arise from impaired motor planning or execution due to musculoskeletal or neurological dysfunction [10]. In individuals with KOA, such alterations may reveal functional deterioration not captured by standard performance-based tests [10].

Alongside advances in biomechanical evaluations of joint function, osteoarthritis (OA) biomarkers, including structural cartilage and inflammation-related proteins, have been extensively investigated for diagnostic and prognostic purposes [[11], [12], [13]]. Yet, none has been implemented in clinical practice. In recent years, proteomic and multi-omics techniques have been employed, which hold promise for uncovering molecular pathways driving OA progression [14,15]. From a clinical perspective, biomarkers should be associated with patient characteristics and relevant outcome measures, and be applicable in routine healthcare settings [16]. Previous studies have related protein levels to demographic features such as age, sex, BMI, and radiological assessment [17], while others have included patient-reported outcome measures (PROMs) of pain [18]. Few studies have combined the molecular aspects of disease progression and their relationship to objectively evaluated joint function [19]. Correlations between performance-based measures of joint function and PROMs are generally moderate to poor and are considered complementary, as they evaluate different constructs [20]. Both measures are therefore relevant for evaluating individuals with KOA [21]. Furthermore, as new biomechanical metrics emerge, their potential clinical utility will depend on demonstrating associations with patient-relevant outcomes, including PROMs [9].

Pain is a hallmark symptom of KOA and a complex, multifactorial experience influenced by peripheral and central neurological pathways [22]. Its intensity often shows little correlation with radiographic features, and recent evidence suggests that pain level and radiographic severity poorly predict functional decline or reduced physical activity [23]. Nevertheless, pain remains an important symptom and a key aspect of the disease experience [22]. Pain mechanisms in KOA are diverse: structural joint changes such as cartilage degradation, osteophyte formation, and synovial inflammation contribute to nociceptive pain, while central sensitisation drives persistence and amplification in many individuals [22]. Inflammation further modulates nociceptive processing, both peripherally and centrally [22]. A deeper understanding of these interactions is needed to clarify their relationship with pain and functional outcomes in KOA.

Against this background, we employed targeted proteomics to characterise plasma proteins involved in inflammatory and neurobiological pathways, combining this molecular profiling with objectively evaluated knee function and PROMs to investigate their associations. By incorporating movement smoothness as a granular biomechanical measure, together with biomarkers and PROMs, our objective was to detect subtle yet clinically meaningful impairments not captured by conventional functional metrics. This exploratory analysis specifically aimed to (1) identify differentially expressed proteins (DEPs) in individuals with KOA compared with controls and (2) examine associations between DEPs, objectively evaluated joint function, including movement smoothness quantified by SPARC, and PROMs to identify potential biomarkers related to impaired joint function or pain.

## 2 Methods

### 2.1 Study design and study participants

This cross-sectional study was conducted in accordance with the Declaration of Helsinki, and ethical approval was obtained from the Swedish Ethical Review Authority (Dnr: 2019–02009). The Strengthening the Reporting of Observational Studies in Epidemiology (STROBE) guidelines were followed [24]. The present study is based on data collected within the BIOFUNC project. BIOFUNC aims to deepen our understanding of KOA through a comprehensive, data-driven approach that integrates proteomics, performance-based function, and patient-reported outcome measures. All participants received verbal and written information about the study procedures and provided written informed consent before participation. Data were collected between March 2021 and January 2024.

Thirty-six individuals with symptomatic unilateral KOA were recruited from two orthopaedic departments and an outpatient clinic in Stockholm, Sweden. The inclusion criteria were KOA diagnosis according to the American College of Rheumatology guidelines [25], ability to repeatedly walk 10 m unaided, and ability to communicate in Swedish or English. Exclusion criteria were predominant pain from sites other than the affected knee, prior joint replacement or intra-articular fracture of the lower limb, rheumatoid arthritis, and/or neurological disorders. Nineteen individuals without musculoskeletal pain or any musculoskeletal or neurological disorders were recruited through advertisements. These individuals are referred to as controls. The controls were frequency-matched for age and sex to individuals with KOA.

### 2.2 Procedures

All data, except radiological examinations, were collected during one session at the Motion Analysis Laboratory at the Karolinska University Hospital, Stockholm, Sweden. Each session followed an identical protocol for both groups and included, in the following order, a structured interview, performance-based functional tests monitored with a sensor-based motion analysis system, completion of PROMs and venous blood sampling. Testing sessions were conducted throughout the day (morning and afternoon), with a similar distribution across groups. Samples were centrifuged at 1440 g for 10 min, and plasma was frozen in aliquots within 2 h of sampling and stored at −80 °C until analysis.

### 2.3 Objectively evaluated joint function

All participants performed four performance-based functional tests, as described in detail elsewhere [26]. The tests included 10 m Fast-Paced Walk (10MWT), 30 s Sit-to-Stand (30STS), Single Leg Mini Squat (SLMS) [27,28], and maximal Jump for Distance (JFD) [29]. For 10MWT, the fastest of the two trials was used for further analysis. The maximum number of sit-to-stand repetitions and SLMS for each leg in 30 s were recorded. The maximal jump distance, measured in centimetres, was recorded as the distance between the starting line and toes after landing (nearest to the starting line).

### 2.4 Knee joint kinematics

Knee joint kinematics during 30STS and SLMS tests were recorded using seven wireless inertial measurement units (Opal, APDM, Portland, OR, USA) [30] (Supplementary Material). Sensors containing triaxial accelerometers, gyroscopes, and magnetometers were positioned on the lumbar region, both thighs, shanks, and feet, following manufacturer's placement guidelines. Data were sampled at 124 Hz, and before each trial (each performance-based test) participants underwent a standardised upright calibration. In the control group, the dominant leg (defined as the preferred leg for kicking a ball) and the non-dominant leg did not differ in the outcome measures; therefore, kinematic data from the affected limb in the KOA group and from the dominant leg, selected as the predefined index limb in the control group, were extracted using Moveo Explorer software (APDM, Portland, OR, USA), which computes joint angles using APDM's quaternion-based orientation estimation and an automated kinematic model. All trials were visually inspected to identify potential signal drift or sensor crosstalk. Sagittal-plane knee flexion/extension angles across the full test duration were processed using a custom MATLAB script (R2025a; The MathWorks, Natick, MA, USA) that segmented repetitions, calculated mean ROM, within-participant ROM variability, and derived angular velocity profiles.

Movement smoothness was quantified using the Spectral Arc Length (SPARC) metric [10], computed from sagittal-plane knee angular velocity. SPARC is a mathematically validated measure of smoothness that is sensitive, reliable, and independent of movement amplitude and duration, making it more robust than traditional jerk-based metrics [10]. Mean SPARC was used to quantify overall movement quality, while SPARC variance was used to quantify movement consistency. These metrics were used in subsequent analyses. Although the application of SPARC to 30STS and SLMS in KOA was exploratory in the present study, the metric has previously demonstrated clinical and construct validity in other populations, including stroke and Parkinson's disease [31,32].

### 2.5 Patient-reported outcome measures

All participants rated current knee pain on a Visual Analogue Scale (VAS, 0–100 mm) directly after performing the functional tests. In addition, individuals with KOA completed the Knee Injury and Osteoarthritis Outcome Score (KOOS) [33], the Forgotten Joint Score-12 (FJS) [34], and the Pain-O-Meter (POM) [35]. The KOOS reliably captures both baseline function and changes over time in individuals with KOA [36]. It consists of five subscales: Pain, Symptoms, Function in Activities of Daily Living (ADL), Function in Sport and Recreation (Sport/Rec), and Knee-Related Quality of Life (QOL), all demonstrating adequate test–retest reliability [37]. Each subscale is scored from 0 to 100, where 0 represents the worst and 100 the best outcome [33]. The FJS is a valid and reliable questionnaire developed to assess outcomes after knee arthroplasty, with final scores ranging from 0 (worst) to 100 (best) [34]. The POM is a validated tool for assessing pain in individuals with various chronic diseases [38]. The instrument has two parts, with the present study using one of them, POM-Words, consisting of 12 sensory and 11 affective words to describe pain qualitatively. An intensity score, unknown to the respondent, is assigned to each word and ranges from 1 to 5, where 1 indicates milder pain than 5. The scores are summed separately for sensory words and affective words. Individuals were instructed to select as many words as needed from both groups to describe their knee pain.

### 2.6 Targeted plasma proteomics

Blood samples collected during the study were analysed using the Olink Target Inflammation and Neuro Exploratory panels, each comprising 92 proteins selected for their relevance to inflammatory and neurobiological pathways. Analyses were performed at the Affinity Proteomics unit, SciLifeLab, Stockholm, Sweden, following the manufacturer's standard protocols and quality control procedures. The platform utilises Proximity Extension Assay (PEA) technology, allowing high-throughput, multiplexed protein quantification with high specificity and sensitivity [39]. Data were returned as Normalised Protein Expression (NPX) values and subjected to statistical analysis. Proteins with NPX values below the limit of detection in more than 80% of the samples were removed. DEPs between groups were defined as ΔNPX greater than 1 (fold change >2) and an adjusted p value less than 0.05. Adjusted p values were calculated using the Benjamini, Krieger, and Yekutieli method to control the false discovery rate at 5% within each comparison.

### 2.7 Radiological examination

Weight-bearing anteroposterior radiographs of the tibiofemoral joint (knees extended), obtained within six months of baseline testing, were graded according to the modified Kellgren–Lawrence (KL) classification (grades 1–4) [40]. Radiographs with KL grades 3–4 were further subclassified by incorporating scores of joint space narrowing (JSN) and bone attrition [41]. A KL grade 3 radiograph with mild JSN was graded as 3a and radiographs with more severe JSN as 3b. A KL grade 4 radiograph with complete loss of joint space was designated 4a if no bone attrition was present, and 4b if subchondral bone attrition was observed. Three assessors classified all radiographs by consensus.

### 2.8 Statistical analysis

Data were analysed using SPSS Statistics (version 26; IBM, Armonk, NY, USA) and GraphPad Prism (version 9). Statistical significance was set at p = .05. Data normality was assessed using Q–Q plots and the Shapiro–Wilk test. Descriptive statistics were presented as mean, standard deviation, frequency, median, and range. Differential protein expression between individuals with KOA and controls was assessed using multiple unpaired t tests, correcting for multiple comparisons via the two-stage step-up false discovery rate method of Benjamini, Krieger, and Yekutieli. Hierarchical cluster analysis (HCA) and principal component analysis (PCA) were performed using heatmapper.ca. Exploratory correlation analyses examined associations between DEPs, clinical outcome measures (performance-based tests and PROMs), and knee kinematics, using Pearson's or Spearman's tests depending on data distribution. Correlation coefficients and nominal p values are reported, with strength interpreted according to Walters, Campbell and Machin: |r| < 0.2 very weak, 0.2 ≤ |r| < 0.4 weak, 0.4 ≤ |r| < 0.6 moderate, 0.6 ≤ |r| < 0.8 strong, and |r| ≥ 0.8 very strong [42].

## 3 Results

### 3.1 Reduced function and increased knee pain in individuals with KOA

Thirty-six individuals with KOA and 19 controls were included; clinical characteristics are summarised in Table 1. Compared with controls, participants with KOA completed fewer repetitions during 30STS and SLMS, took longer to complete 10MWT, and jumped a shorter distance (Table 2). Kinematic analysis demonstrated no between-group differences in knee range of motion for either 30STS or SLMS. However, movement smoothness, quantified using SPARC, demonstrated significant group differences: mean SPARC was lower in the KOA group compared to controls during both 30STS and SLMS, reflecting reduced overall movement quality. In addition, SPARC variance was higher in the KOA group during SLMS, indicating less consistent smoothness across repetitions. Individuals with KOA also reported higher current knee pain on VAS (Table 2).

**Table 1:** Baseline characteristics of study participants with knee osteoarthritis and controls.

Baseline characteristics	KOA group (n = 36)	Control group (n = 19)	Group differences	
			p value	95% CI
Women, n (%)	27 (75)	12 (63)	.370	

	**Mean (SD)**			

Age	62.7 (10.8)	62.3 (10.6)	.882	−5.67 to 6.58
Height, cm	172 (11.1)	172 (10.1)	.997	−6.08 to 6.13
Weight, kg	75.2 (14.1)	71.9 (10.3)	.375	−4.08 to 10.68

	**Median (IQR)**			

Body Mass index, kg/m^2^	24.5 (4.1)	23.7 (2.7)	.250	−0.58 to 2.32

	**Number (%)**			

Previous knee arthroscopy, n (%)	18 (50)	–	–	
**Use of analgesics**				
Never n (%)	22 (61)	–	–	
Sometimes n (%)	9 (25)	–	–	
Daily n (%)	5 (14)	–	–	
**Modified Kellgren-Lawrence score (0–4)**^a^ n (%)				
0	1 (3)	–	–	
1	8 (23)	–	–	
2	2 (6)	–	–	
3a	5 (15)	–	–	
3b	9 (27)	–	–	
4a	8 (23)	–	–	
4b	1 (3)	–	–	

KOA, Knee osteoarthritis.

a = missing data n = 2.

**Table 2:** Performance-based knee function, and patient reported knee function and pain in individuals with knee osteoarthritis and controls.

Performance-based function	KOA group (n = 36)	Control group (n = 19)	Group differences	
			p value	95% CI
10MWT, seconds, mean (SD)	6.4 (1.6)	5.3 (0.7)	.006	0.27 to 1.81
30STS, n of repetitions, mean (SD)	15 (5)	21 (7)	**<.001**	−9.26 to −2.70
SLMS, n of repetitions, mean (SD)	20 (10)	36 (10)	**<.001**	−22.19 to −8.96
JFD, cm, mean (SD)	121 (38)^a^	145 (45)	**.044**	−47.28 to −0.66
**Knee kinematics during performance-based tests**				
Mean ROM 30STS, degrees, mean (SD)	77.3 (10.7)^a^	80.2 (9.1)^a^	.228	−7.68 to 1.88
Mean SPARC 30STS, median (IQR)	−11.2 (0.33)^a^	−10.9 (0.35)^b^	**.004**	−0.342 to −0.085
SPARC variance 30STS, median (IQR)	0.005 (0.014)^a^	0.003 (0.003)^b^	.052	−8 × 10^−6^ to 5 × 10^−3^
Mean ROM SLMS, degrees, mean (SD)	38.1 (13.3)	43.3 (11.1)	.149	−12.37 to 1.94
Mean SPARC SLMS, mean (SD)	−11.6 (0.38)	−11.2 (0.24)	**<.001**	−0.64 to −0.25
SPARC variance SLMS, median (IQR)	0.015 (0.04)^a^	0.003 (0.01)^b^	**<.001**	0.002 to 0.021

**Patient-reported outcome measures**	**Median (min-max)**			

**Current knee pain, visual analogue scale** (0–100 mm)	20 (0–57)	0 (0–11)^b^	**<.001**	
**KOOS** (0–100; worst-best)				
Pain	72 (36–100)	–	–	
Symptoms	64 (21–100)	–	–	
Function in ADL	85 (50–100)	–	–	
Function in sport and recreation	43 (5–100)	–	–	
Knee-related quality of life	47 (19–94)	–	–	
**Forgotten joint score-12**(0–100; worst-best)	32 (0–96)	–	–	
**Pain-o-Meter**				
Sensory words, intensity value (0–36)	7 (0–31)	–	–	
Affective words, intensity value (0–42)	6 (0–19)	–	–	

10MWT, 10 m Fast-Paced Walk test; 30STS, 30 s Sit-to-Stand test; SLMS, Single Leg Mini Squat test; JFD, Maximal Jump for Distance; ROM, Range of Motion; SPARC, Spectral Arc Length; KOOS, Knee Injury and Osteoarthritis Outcome Score; ADL, Activities of Daily Living.

a = missing data n = 2.

b = missing data n = 1.

### 3.2 Altered plasma profile in individuals with KOA compared to controls

**Fig. 1:**
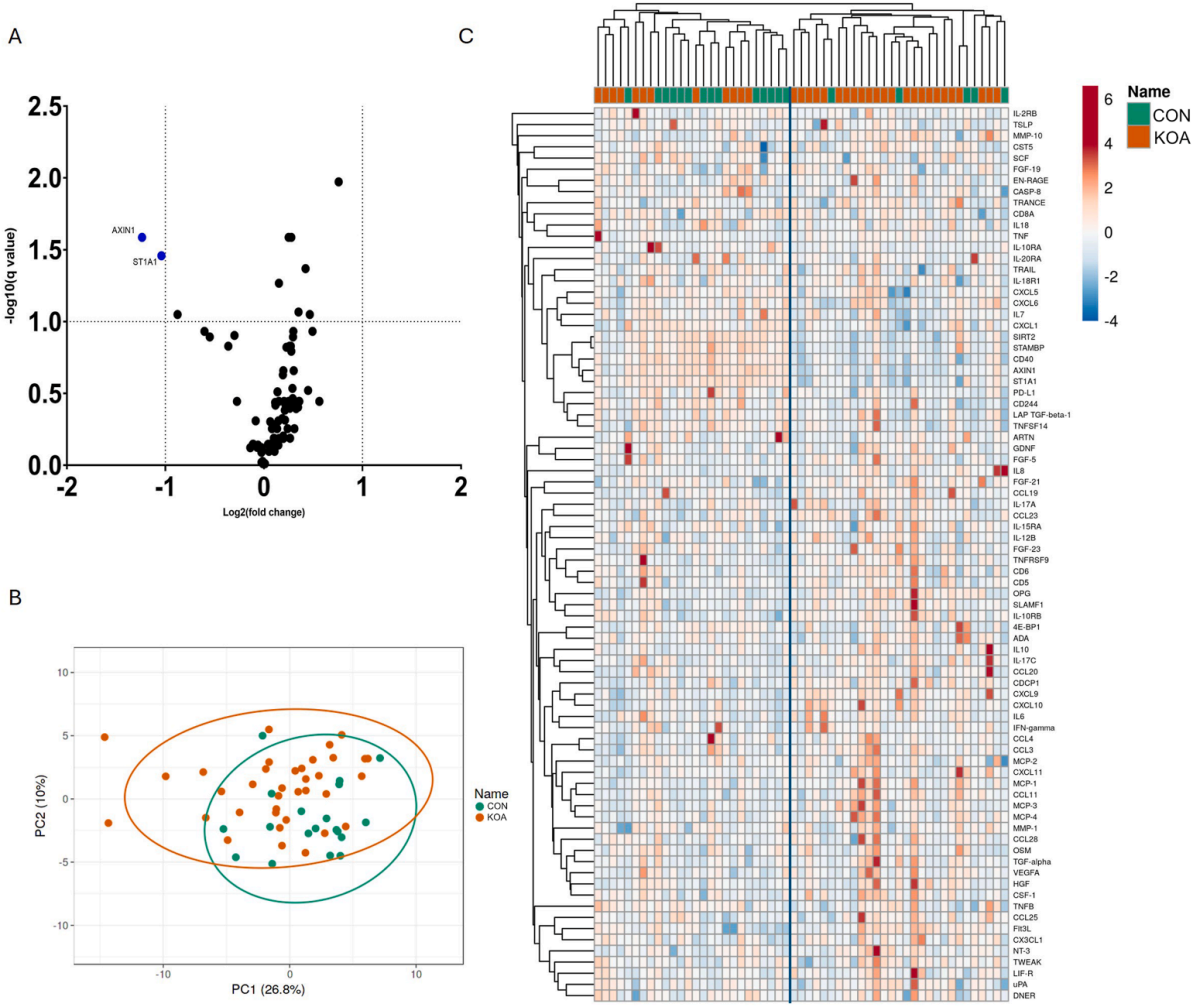
(A) Volcano plot displaying differentially expressed proteins from the inflammation panel in individuals with KOA compared with controls. Principal component analysis (B) suggested partial separation between the KOA and control groups, with overlap between groups. Similarly, hierarchical cluster analysis (C) showed a tendency for samples to cluster by group, although overlap between individuals with KOA and controls was observed based on their protein expression patterns. CON, controls; KOA, individuals with knee osteoarthritis.

**Fig. 2:**
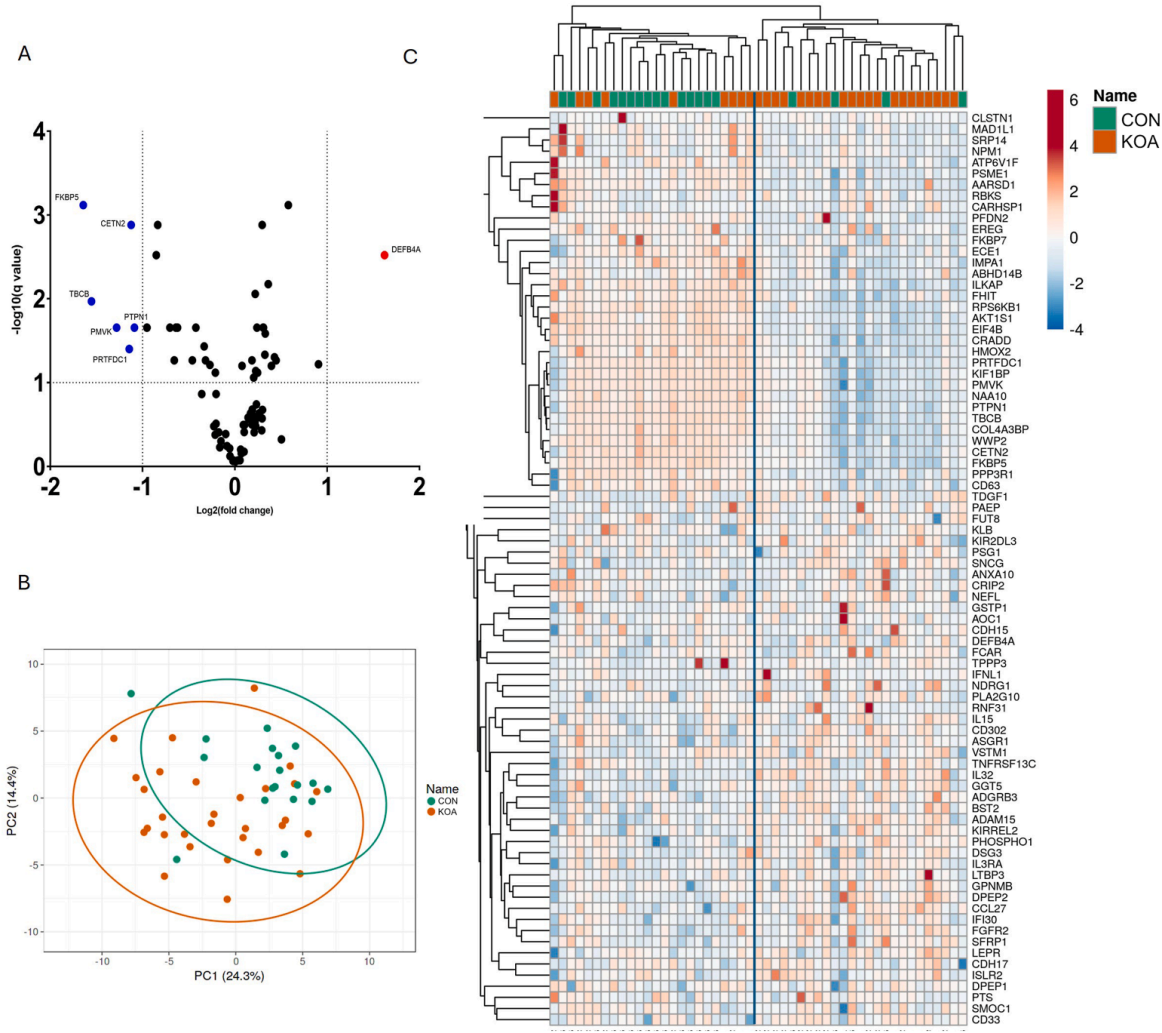
(A) Volcano plot displaying differentially expressed proteins from the neuro exploratory panel in individuals with KOA compared with controls. Principal component analysis (B) suggested partial separation between the KOA and control groups, with overlap between groups. Similarly, hierarchical cluster analysis (C) showed a tendency for samples to cluster by group, although overlap between individuals with KOA and controls was observed based on their protein expression patterns. CON, controls; KOA, individuals with knee osteoarthritis.

In the plasma samples from individuals with KOA and controls, we profiled inflammation- and neuro-related proteins using the Olink Inflammation and Neuro Exploratory panels. 82 of 92 proteins from the neuro exploratory panel, and 80 of 92 proteins from the inflammation panel were detected. Nine DEPs were identified: two from the inflammation panel (Fig. 1A) and seven from the neuro exploratory panel (Fig. 2A). From the inflammation panel Sulfotransferase 1A1 (ST1A1) and Axin-1 (AXIN1), were downregulated in individuals with KOA (Fig. 1A). From the neuro exploratory panel β-defensin 4A (DEFB4A) was upregulated, whereas FK506-binding protein 5 (FKBP5), Centrin-2 (CETN2), Tubulin-folding cofactor B (TBCB), Phosphomevalonate kinase (PMVK), Protein tyrosine phosphatase, non-receptor type 1 (PTPN1) and Phosphoribosyl transferase domain containing 1 (PRTFDC1) were downregulated compared to controls (Fig. 2A). PCA suggested partial separation between KOA and control samples, with notable overlap (Fig. 1B and 2B). Similarly, HCA demonstrated stronger group-level clustering, with most KOA and control samples forming separate clusters, but with several samples positioned in the opposite groups (Fig. 1C and 2C).

### 3.3 Associations between DEPs, objectively evaluated joint function, and PROMs

**Fig. 3:**
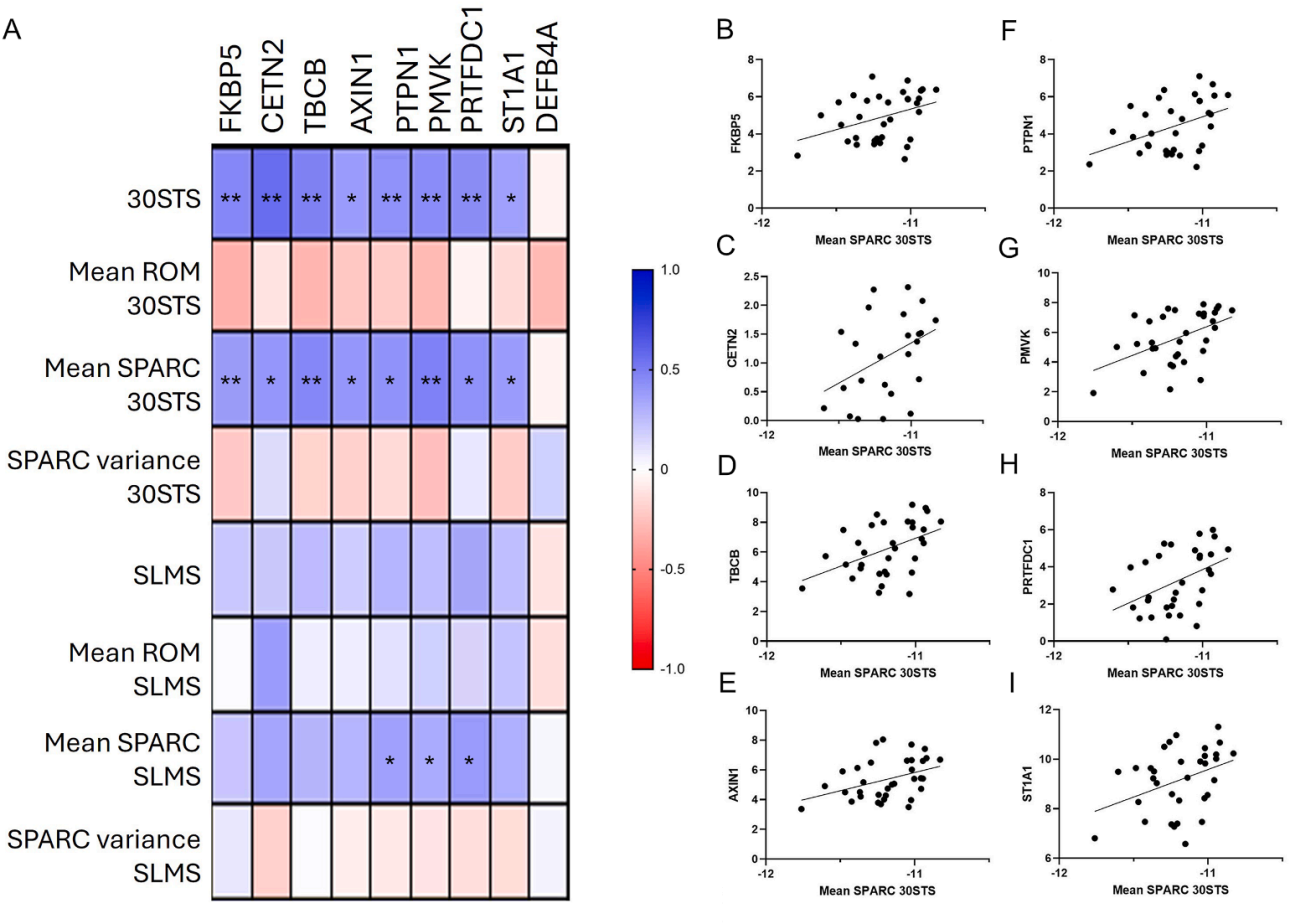
(A) Colour matrix showing the associations in the KOA group between the 30 s Sit-to-Stand test (30STS), Single Leg Mini Squat test (SLMS), and knee kinematics during these tests, and both upregulated and downregulated DEPs. Correlations meeting the p value threshold p ≤ .05 are marked ∗, and those with p ≤ .01 are marked ∗∗. (B–I) Correlations in the KOA group between mean SPARC during the 30STS and the downregulated DEPs: (B) FKBP5, FK506-binding protein 5; (C) CETN2, Centrin-2; (D) TBCB, Tubulin-folding cofactor B; (E) AXIN1, Axin-1; (F) PTPN1, Protein tyrosine phosphatase, non-receptor type 1; (G) PMVK, Phosphomevalonate kinase; (H) PRTFDC1, Phosphoribosyl transferase domain containing 1 and (I) ST1A1, Sulfotransferase 1A1.

To investigate associations between the potential biomarkers identified from the molecular profiling and clinical characteristics, correlation analyses were performed between the DEPs, clinical outcomes (performance-based tests and PROMs), and objective biomechanical measures of knee kinematics (Fig. 3A, Supplementary Table 1). All downregulated proteins demonstrated weak-to-moderate positive correlations with 30STS, while three (CETN2, PMVK, and ST1A1) showed weak-to-moderate negative correlations with 10MWT (Supplementary Table 1). Mean SPARC values, representing movement smoothness, showed weak-to-moderate positive correlations with all downregulated proteins in 30STS (Fig. 3B–I) and with three of them in SLMS (Supplementary Table 1).

Analysis of associations between PROMs and joint function revealed that VAS correlated positively with SLMS SPARC variance. KOOS subscales (ADL, Pain, Sport and Recreation), FJS, and the POM affective dimension showed associations with various performance-based tests and kinematic measures (Supplementary Table 1). The POM sensory subscale correlated positively with PTPN1 and PRTFDC1 (Supplementary Table 2).

Correlation analysis among DEPs demonstrated strong positive associations between all downregulated proteins (r = 0.89–0.97), whereas DEFB4A showed weak correlations with the other proteins (r = 0.05–0.10), indicating a distinct expression pattern (Supplementary Fig. 1).

## 4 Discussion

This exploratory study provides new insights into the molecular and functional features of individuals with KOA, emphasising the value of integrating proteomic data with clinical outcomes (performance-based tests and PROMs) and objective biomechanical measures of knee kinematics, including the novel application of the movement smoothness metric SPARC. By identifying nine DEPs and observing associations between eight of them to performance-based tests as well as to movement smoothness, the study highlights potential biological pathways that may contribute to functional impairment in KOA. Such integration may provide a basis for future efforts to define patient subgroups and enable more targeted treatment [43]. Overall, moderate associations indicate that these assessment domains capture related but distinct KOA aspects.

In line with previous findings, individuals with KOA reported significantly higher knee pain levels and performed worse in all tests than controls [26]. Performance differences were observed in general mobility tasks, such as 10MWT, and more demanding neuromuscular tasks, such as 30STS and JFD. This pattern implies a multifactorial impairment in functional performance, likely influenced by pain, muscle weakness, and altered motor function. In addition to these performance deficits, individuals with KOA also demonstrated altered movement smoothness, evidenced by lower mean SPARC values and higher SPARC variance during functional tasks.

At the molecular level, we analysed the proteomic profile in individuals with KOA, and identified nine differentially expressed proteins compared with controls. One protein, β-defensin 4A (DEFB4A), was upregulated in individuals with KOA. β-Defensins belong to a family of antimicrobial peptides that are key effectors of innate immunity [44,45]. Beyond host defence, they are increasingly recognised as modulators of inflammatory processes in non-infectious diseases, including OA [[44], [45], [46]]. Previous studies have shown that mesenchymal tissues, including synovial membrane and cartilage, can produce β-defensins in response to pro-inflammatory stimuli [44,45]. These molecules may be upregulated by transforming growth factor-β, interleukin (IL) 1, and tumour necrosis factor (TNF), which are involved in OA pathogenesis [44,45]. One study hypothesised that β-defensins contribute to OA progression by enhancing catabolic pathways in the articular cartilage, leading to extracellular matrix degradation and joint degeneration [46]. Therefore, its upregulation in our KOA cohort may reflect inflammatory and tissue remodelling activity, that could contribute to cartilage breakdown and disease progression [44].

Eight proteins were downregulated in individuals with KOA compared to controls. Although their specific roles in OA are not fully understood, many have previously been linked to pathways involved in inflammation, cellular stress response, cytoskeletal organisation, metabolic regulation, and cell signalling [[47], [48], [49], [50], [51], [52]]. Among them, FKBP5 is of particular interest, as transcriptomic and histological studies have suggested its involvement in OA [[47], [48], [49]]. FKBP5 is a scaffold immunophilin protein that modulates glucocorticoid receptor (GR) sensitivity and regulates stress and inflammatory processes. It has been reported as downregulated in osteoarthritic synovial and cartilage tissues, with increased methylation suggesting epigenetic suppression [47,48]. Reduced FKBP5 expression may therefore reflect impaired GR-mediated anti-inflammatory regulation and altered stress response within OA-affected joints. In contrast, increased FKBP5 immunoreactivity has been observed in synovium, cartilage, and Hoffa's fat pad of a homogeneous group of 14 individuals with advanced KOA (KL grade 4) and concomitant severe chronic pain and disability, where it co-localised with TNF-α and IL-6, supporting involvement in local inflammatory activity [49].

PTPN1 may also be relevant in this context. A recent study reported increased PTP1B expression, the protein encoded by PTPN1, in OA-damaged cartilage, while experimental PTPN1 knockdown restored chondrocyte homeostasis and attenuated OA-related changes in experimental models [50]. In contrast, PTPN1 was downregulated in plasma in our KOA group. This discrepancy does not necessarily indicate conflicting biology, as protein expression in diseased joint tissues and circulating plasma reflects different biological compartments and may capture distinct aspects of OA pathophysiology, including local tissue responses and systemic molecular alterations.

Taken together, downregulation of FKBP5, PTPN1, and other proteins may indicate broader molecular disturbances contributing to joint degeneration and symptoms. The downregulated proteins were highly correlated with each other, whereas DEFB4A showed little association with the remaining markers, indicating a distinct expression pattern. Although causality cannot be established, these alterations may reflect disrupted cellular homeostasis and contribute to the biological heterogeneity of KOA. Further studies are warranted to determine their potential role in disease progression or secondary responses to other pathological processes.

The consistent positive associations between all eight downregulated proteins and 30STS, and the negative associations of three of them with 10MWT, suggest that these molecular alterations may be associated with functional performance. While standard tests quantify outcomes such as time, distance, or number of repetitions [9], movement smoothness analysis provides additional information on the quality of movement execution, serving as an indirect proxy for motor control [10,31,32]. The observed impairments imply that KOA affects not only the capacity to generate force or complete tasks but also the quality and consistency of movement execution. Interestingly, we also found associations between this metric and DEPs. Specifically, higher mean SPARC values, indicating smoother movement, were associated with higher expression levels of all downregulated proteins in 30STS and with three of them in SLMS. These findings together suggest a potential relationship between protein expression and objectively assessed movement quality. As these correlations were exploratory and uncorrected for multiple comparisons, they require independent confirmation.

Recent reviews on biomechanics in OA, nearly 80% of the reviewed studies focused on the knee [7,8]. Direct, sensor-derived measurements such as IMUs were applied in only about 9% of all studies analysed in these reviews, indicating they remain underused in OA research [7,8]. Our study demonstrates their value by showing that IMU-derived data can capture impairments in movement quality that conventional kinematics, such as ROM, cannot.

OA is associated with chronic, low-grade systemic and local inflammation, which can disrupt the balance between anabolic and catabolic processes within the joint [53]. Consequently, extensive research has focused on inflammatory mediators, such as cytokines and chemokines [54]. Several molecules implicated in OA initiation and progression, such as IL-6, IL-8, IL-15, TNF, and C–C motif chemokine ligand 2 (CCL2), were included in the Olink panels used in our study. Surprisingly, our analysis did not reveal any significant differences in the levels of these selected proteins between individuals with KOA and controls. It is difficult to determine whether this finding is due to the limited sample size, the possibility that the included participants with KOA were at a disease stage in which systemic cytokine levels were not markedly elevated, and/or low-grade inflammation typical of OA that may not be detectable in plasma samples. This suggests a role for alternative inflammatory processes in KOA, beyond the cytokines and inflammation-related proteins targeted here [53,54].

In this study, we applied a range of PROMs to assess different aspects of the patient experience and health-related quality of life, including pain, daily function, sport and recreation, joint awareness, and emotional aspects of pain. Despite addressing distinct constructs, several instruments showed moderate correlations with performance-based functional outcomes. These associations support the validity of the battery of functional tests used in this study by demonstrating their relevance to patient-perceived symptoms and limitations. Moreover, the consistent direction of correlations supports the complementary role of PROMs and objective assessments, confirming that combining these approaches provides a more comprehensive understanding of disease impact in KOA.

This broader integrative perspective is also reflected in the work of Mündermann et al. [19], who related load-induced serum biomarker changes to accumulated knee load and PROMs in individuals with medial compartment KOA. Their study used a 30 min walk as a loading stimulus rather than as a performance measure and thus did not establish a direct connection between standardised performance-based tests and biochemical biomarker profiles. At present, these two domains have largely progressed independently, with biomarkers capturing molecular and mechanically driven joint processes and functional tests reflecting clinical capability. The limited intersection highlights a critical knowledge gap that our study seeks to address by integrating molecular and functional approaches to advance understanding of KOA.

Few associations were identified between pain-related PROMs and functional tests or DEPs. Higher VAS scores correlated with greater SPARC variance in SLMS, indicating that pain may affect movement consistency in KOA. No associations were found between VAS scores and DEPs. However, despite being limited to one PROM, the positive associations between the POM sensory subscale and PTPN1 and PRTFDC1 may suggest a molecular link between pain perception and protein expression in KOA.

The study has several important strengths. We applied a multidimensional approach to better capture the complexity of the disease, integrating targeted serum proteomics with clinical outcomes and objective biomechanical measures. The comprehensive assessment battery ensured robust clinical characterisation, and the omics approach using PEA technology provided reliable molecular insights. Notably, we identified nine DEPs, eight of which showed associations with movement smoothness [10,31,32], highlighting depth and innovation of the current approach. These strengths should be considered alongside limitations. This study should be interpreted within the scope of its exploratory, cross-sectional design. The sample was relatively small, limiting statistical power and generalisability, and the cross-sectional design precludes causal inference. The low BMI of the KOA cohort limits generalisability to more advanced or obese populations, although it reduces confounding from obesity-related factors. Although dominant and non-dominant limbs did not differ in controls, a small bias related to limb selection between groups cannot be excluded and may have influenced the magnitude of some between-group differences. The correlation analyses and the application of movement smoothness (SPARC) were exploratory and uncorrected for multiple comparisons, so these associations are hypothesis-generating and the risk of false-positive findings should be borne in mind. The use of targeted panels means other potentially relevant proteins were not captured. Finally, as blood sampling followed functional testing rather than a standardised resting period, we cannot exclude an influence of acute loading on protein levels, although the brief, low-load protocol makes a substantial effect unlikely.

In conclusion, our findings support the potential of combining targeted proteomics with objective biomechanical measures and clinical outcomes to reveal biologically and clinically relevant patterns in KOA. This integrative approach may help bridge molecular findings with functional outcomes and, ultimately, support the development of more personalised and timely treatment strategies.

## Author contributions

JEN, CA, MBS: Conception and study design; JEN, CA, KG: data collection; JEN, CA, MBS, KG, MH: data and statistical analysis; JEN, CA, MBS, KG: interpretation of data, JEN, CA, MBS, KG: drafting the manuscript; All authors: participated in critical revision of the manuscript; JEN, CA: Funding; All authors read and approved the final submitted manuscript.

## Declaration of generative AI in scientific writing

During the preparation of this work the author (KG) used Paperpal in order to improve the consistency and grammar of this manuscript. After using this tool/service, the author reviewed and edited the content as needed and takes full responsibility for the content of the publication.

## Funding

This study was supported by grants provided by *Strategiska forskningsområdet vårdvetenskap* (SFO–V), Karolinska Institutet; Alex och Eva Wallströms stiftelse; Magnus Bergvalls stiftelse; af Ugglas stiftelse; Stiftelsen Skobranschens utvecklingsfond; Loo och Hans Ostermans stiftelse; Åke Wibergs stiftelse; and Angeby stiftelse. Josefine E. Naili was supported by Region Stockholm (clinical research appointment). Morten Bilde Simonsen was supported by the 10.13039/501100009708Novo Nordisk Foundation, Denmark (grant no. NNF21OC0065373). The funders had no role in the study design, data collection and analysis, interpretation of data, decision to publish, or preparation of the manuscript.

## Conflict of interest

Each author certifies that there are no commercial associations that might pose a conflict of interest in connection with the submitted article.
