# Supplementary material for: Movement smoothness and plasma proteomics are associated with functional impairment in knee osteoarthritis
Source: Osteoarthr Cartil Open. 2026 Jul 30;8(3):100854. doi: 10.1016/j.ocarto.2026.100854 (PMC13472034; doi:10.1016/j.ocarto.2026.100854)
Supplement: Supplementary file 1 [file mmc2.pdf]

Supplementary Table 1. Summary of correlations between proteomic biomarkers (DEPs) and patient-reported outcome measures (PROMs) with performance-based tests and knee kinematics.

		Differentially Expressed Proteins (DEPs)									Patient-reported outcome measures (PROMs)								
		downregulated								upregulated	KOOS					FJS	POM		VAS
		FKBP5	CETN2	TBCB	AXIN1	PTPN1	PMVK	PRTFDC1	ST1A1	DEFB4A	Pain	Symptoms	ADL	Sport/Rec	QOL		sensory	affective	
Performance-based tests	10MWT		r = −0.400 p = 0.043				r = −0.364 p = 0.032		r = −0.372 p = 0.028				r = −0.444 p = 0.007	r = −0.410 p = 0.013		r = −0.346 p = 0.039			
	JFD												r = 0.413 p = 0.015						
	SLMS										r = 0.333 p = 0.047		r = 0.468 p = 0.004						
	30STS	r = 0.456 p = 0.006	r = 0.567 p = 0.002	r = 0.480 p =0.004	r = 0.374 p = 0.025	r = 0.429 p = 0.010	r = 0.445 p = 0.007	r = 0.442 p = 0.009	r = 0.368 p = 0.030				r = 0.403 p = 0.015						
Knee kinematics	Mean ROM 30STS																		
	Mean SPARC 30STS	r = 0.377 p = 0.006	r = 0.405 p = 0.050	r = 0.455 p =0.008	r = 0.397 p = 0.020	r = 0.412 p = 0.017	r = 0.488 p = 0.004	r = 0.426 p = 0.015	r = 0.375 p = 0.032		r = 0.362 p = 0.035		r = 0.364 p = 0.034	r = 0.355 p = 0.040				r = −0.418 p = 0.014	
	SPARC variance 30STS																		
	Mean ROM SLMS												r = 0.375 p = 0.024						
	Mean SPARC SLMS					r = 0.367 p = 0.030	r = 0.334 p = 0.050	r = 0.394 p = 0.021			r = 0.359 p = 0.031		r = 0.395 p = 0.017						
	SPARC variance SLMS										r = −0.386 p = 0.020							r = 0.337 p = 0.044	r = 0.403 p = 0.015

Abbreviations: 10MWT, 10-m Fast-Paced Walk test; JFD, Maximal Jump for Distance; SLMS, Single Leg Mini Squat test; 30STS, 30-s Sit-to-Stand test; ROM, Range of Motion; SPARC, Spectral Arc Length; FKBP5, FK506-binding protein 5; CETN2, Centrin-2; TBCB, Tubulin-folding cofactor B; AXIN1, Axin-1; PTPN1, Protein tyrosine phosphatase, non-receptor type 1; PMVK, Phosphomevalonate kinase; PRTFDC1, Phosphoribosyl transferase domain containing 1; ST1A1, Sulfotransferase 1A1; DEFB4A, β-defensin 4A; KOOS, Knee Injury and Osteoarthritis Outcome Score; ADL, Function in Activities of Daily Living; Sport/Rec, Function in Sport and Recreation; QOL, Knee-Related Quality of Life; FJS, Forgotten Joint Score-12; POM, Pain-O-Meter; VAS, Visual Analogue Scale.
