# Supplementary material for: Movement smoothness and plasma proteomics are associated with functional impairment in knee osteoarthritis
Source: Osteoarthr Cartil Open. 2026 Jul 30;8(3):100854. doi: 10.1016/j.ocarto.2026.100854 (PMC13472034; doi:10.1016/j.ocarto.2026.100854)
Supplement: Supplementary file 2 [file mmc3.pdf]

Supplementary Table 2. Summary of correlations between proteomic biomarkers (DEPs) and patient-reported outcome measures (PROMs).

| | | | Patient-reported outcome measures (PROMs) | | | | | | | | |
|---|---|---|---|---|---|---|---|---|---|---|---|
| | | | KOOS | | | | | FJS | POM | | VAS |
| | | | Pain | Symptoms | ADL | Sport/Rec | QOL | | sensory | affective | |
| Differentially Expressed Proteins (DEPs) | Inflammation panel | AXIN1 | | | | | | | | | |
| | | ST1A1 | | | | | | | | | |
| | Neuro Exploratory panel | FKBP5 | | | | | | | | | |
| | | CETN2 | | | | | | | | | |
| | | TBCB | | | | | | | | | |
| | | PTPN1 | | | | | | | r = 0.377<br>p = 0.025 | | |
| | | PMVK | | | | | | | | | |
| | | PRTFDC1 | | | | | | | r = 0.456<br>p = 0.007 | | |
| | | DEFB4A | | | | | | | | | |

*Abbreviations:* AXIN1, Axin-1; ST1A1, Sulfotransferase 1A1; FKBP5, FK506-binding protein 5; CETN2, Centrin-2; TBCB, Tubulin-folding cofactor B; PTPN1, Protein tyrosine phosphatase, non-receptor type 1; PMVK, Phosphomevalonate kinase; PRTFDC1, Phosphoribosyl transferase domain containing 1; DEFB4A, β-defensin 4A; KOOS, Knee Injury and Osteoarthritis Outcome Score; ADL, Function in Activities of Daily Living; Sport/Rec, Function in Sport and Recreation; QOL, Knee-Related Quality of Life; FJS, Forgotten Joint Score-12; POM, Pain-O-Meter; VAS, Visual Analogue Scale.
