# Supplementary material for: Movement smoothness and plasma proteomics are associated with functional impairment in knee osteoarthritis
Source: Osteoarthr Cartil Open. 2026 Jul 30;8(3):100854. doi: 10.1016/j.ocarto.2026.100854 (PMC13472034; doi:10.1016/j.ocarto.2026.100854)
Supplement: Supplementary file 3 [file mmc1.docx]

**Supplementary Material**

**Sensor Hardware Characteristics**

- Opal sensors record **3D acceleration, angular velocity, magnetic field**, and an onboard **orientation estimate**.
- Typical hardware ranges:
  - Accelerometer: ±2g or ±6g
  - Gyroscope: ±2000°/s
  - Magnetometer: ±6 Gauss
  - Maximum internal sensor sample rate: **1280 Hz**; output rate configurable to **20–128 Hz**.
- Wireless synchronization across sensors is accurate to **10 µs**.

**Kinematic Model & Joint Angle Computation**

- Orientation is represented using **quaternions**, transformed from sensor frame to Earth frame via proprietary manufacturer algorithms.
- Joint angles (e.g., knee flexion/extension) are derived from relative segment orientations using Euler‑angle decomposition implemented within Moveo Explorer.

**Sensor‑Fusion Algorithm**

- APDM uses a **proprietary fusion method**, combining gyroscope integration (time‑update) with accelerometer/magnetometer corrections (measurement‑update) to mitigate drift.
- Magnetometer influence is automatically reduced when magnetic disturbance is detected.

**Preprocessing & Quality Control**

- Quality control was performed on the exported knee-angle signals prior to analysis. Recordings were screened automatically for file integrity, missing or invalid data, and plausibility of repetition detection. Knee-angle signals were low-pass filtered and repetitions were identified using automated peak-detection algorithms. Trials with insufficient or implausible repetition detection were flagged and excluded from per-repetition analyses. Visual inspection of diagnostic plots was additionally performed where needed.
- Raw sensor data undergo **low‑pass filtering** with an approximate **50 Hz bandwidth** before fusion.
