# Supplementary material for: Movement smoothness and plasma proteomics are associated with functional impairment in knee osteoarthritis
Source: Osteoarthr Cartil Open. 2026 Jul 30;8(3):100854. doi: 10.1016/j.ocarto.2026.100854 (PMC13472034; doi:10.1016/j.ocarto.2026.100854)
Supplement: Supplementary file 4 [file mmc4.docx]

**Supplementary Figure 1.** Correlation heatmap showing pairwise associations between differentially expressed proteins (DEPs) in the KOA group. Colors represent correlation coefficients ranging from −1 to +1, with red indicating positive correlations, blue indicating negative correlations, and white indicating no correlation. Strong positive correlations were observed among the downregulated proteins, while the upregulated protein DEFB4A showed weak associations with the other markers.
